# Prevalence and density of malaria parasitaemia amongst HIV Individuals in Warri, Nigeria

**DOI:** 10.4314/ahs.v21i2.16

**Published:** 2021-06

**Authors:** Johnson Daniel Jemikalajah, Clement Oliseloke Anie, Felix Oghenemaro Enwa

**Affiliations:** 1 Department of Microbiology, Faculty of Science, Delta State University Abraka, Delta State, Nigeria; 2 Department of Pharmaceutical Microbiology, Faculty of Pharmacy, Delta State University, Abraka, Delta State, Nigeria

**Keywords:** Density, Malaria, HIV, prevalence, Nigeria

## Abstract

**Background:**

Malaria parasite has been observed to be a common infection in Human Immunodeficiency virus (HIV), an increase malaria infection in adults.

**Objective:**

This experimental study is sets to determine the prevalence and density of malaria parasitaemia in Warri community, South-Southern Nigeria.

**Methods and Results:**

A total of 600 participants were screened for Human immunodeficiency virus and malaria parasite using WHO systems two and Geimsa staining technique for thick and thin blood films and absolute parasite counts done respectively. The prevalence rate of 38% and 39% were obtained for malaria parasite infection among HIVSP and HIV/span>SN respectively. The difference in malaria parasite infection was not statistically significant (P>0.05) between HIVSP and HIVSN. However, the mean parasite density in HIVSP was significant (P<0.05) when compared with HIVSN. The mean parasite densities of 2384 ± 747 and 1883 ± 645 were recorded for HIVSP and HIVSN respectively. The mean parasite densities of 2385 ± 782 and 2383 ± 717 observed for males and females respectively showed no statistical significant difference (P<0.05).

**Conclusion:**

This study has shown a high prevalence of malaria parasite among the HIV infected subjects.

## Introduction

Malaria is a parasitic disease that is transmitted from one individual to another through the bite of the female anopheles mosquito^1^. Those living in the endemic and poorest regions are vulnerable to contracting malaria^2^. It is the commonest disease of the tropical and subtropical regions of the world and accounts for more than 300 million infected persons and a minimum of 1 million deaths yearly^3^. It is an endemic disease in Nigeria and constitutes about 60% of the illness consultations in the country and about 10% in Warri, south south^4^ Nigeria. Malaria accounts for 25–30% of infant and childhood mortality^5^. In Warri, south-south of Nigeria, the malaria parasite rate is 57.9%^6^. Human immunodeficiency virus (HIV) infection has been a major cause of illness and death among children, teens and young adults' worldwide^7^. It accounts for most premature mortality in sub-Saharan Africa and the entire world^8^

In respect to geographical overlap between malaria and HIV/AIDS, a reasonable number of coinfections occur^9^. Infection with HIV-1 causes progressive cellular immunosuppression, and any resulting impairment in immune response to malaria might be associated with failure to prevent infection^10^. Some components of human immune response to *P. falciparum* are modified by HIV and further increase the potential reservoir in the patient thereby increasing the number of chemokine receptor positive macrophages. This response stimulates HIV-1 replication through the production of cytokines by activated lymphocytes^11^. During the process, the number of chemokine receptor positive macrophages was increased as result of elevated reservoir for HIV in the placenta^12^.

A study in Guinea Bissau found no difference in HIV-2 progression as measured by viral load between adults with parasitaemia and those without^13^. Whereas, in Malawi HIV plasma viral load were found to be significantly higher in patient with malaria than in those without^14^. This is slightly difficult to interpret because there was no base line viral load measurement available before the episodes of malaria.HIV infection and malaria are disturbing because they co-exist in African countries^15^,^16^ but studies vary in their outcomes in the interactions between the infections^17^,^18^. This may be why HIV and malaria still remain the most essential global health challenges of the developing countries including Nigeria where it has been reported to cause more than 4 million deaths yearly with HIV infection increasing the danger and severity of malaria infection and its burdens^19^. This suggests that malaria can cause faster progression of HIV disease whereas HIV in turn facilitates the rate of malaria transmission which causes strong immune cell activation and up-regulation of pro-inflammatory cytokines production which create ideal microenvironment for the spread of HIV among CD4 cells, thus rapid HIV replication^20^. This provides the best evidence of an effect of malaria of HIV in non pregnant adults.

Both HIV and malaria are probable factors in the modification of each other's development, disease severity, and disease progression rate^21^. This is an indication that malaria is still a compound factor in HIV infection. Thus, this study was aimed at determining the prevalence and density of Malaria parasitaemia among HIV infected patients to provide information to improve the management of HIV and malaria in

## Materials and Methods

This study was conducted amongst inhabitants of Warri, in Central Hospital, Nigeria for a period of 2016 to 2018. Prospective comparative study was carried at Central Hospital, Nigeria. A total of 600 subjects were recruited into the study.

### Ethical and sample collection

Samples were collected from participants based on the approved ethical procedures of Central Hospital, Warri research and ethics committee with No. CHW/HA/45/2018.

### Sample analysis

Blood samples were collected from the subjects into EDTA containers and transported to the laboratory for analysis.

### Human immunodeficiency virus screening

This was carried out using two enzymes linked immunosorbent assay rapid screening kits based on WHO systems two^22^. Determine and immunocomb II rapid screening kits of Abbott Laboratories, Japan and Organics, France were used. Test was carried out according to manufacturer's instruction.

### Malaria parasite screening

This was determined by Geimsa staining technique of thick and thin blood films16. Malaria parasite count was done using WHO standard method of absolute parasite count (counting parasites number/microlitre of blood)^23^ .

### Data analysis

Data obtained were analyzed using statistical package for social sciences(IBM SPSS version 25.0 which includes descriptive statistics,mean, standard deviation for observed variables. The association between variables was tested using Chi-square and t-test to compare infectivity between seropositive and seronegative subjects. All test were two tails with statistical significance set at 5% (0.05) confidence level.

## Results

Out of the 600 subjects examined, 300 were HIV seropositive (HIVSP), comprising of 143 males and 157 females while 300 were HIV-seronegative (HIVSN).. The prevalence rate of 38% and 39% were obtained for malaria parasite infection amongst HIVSP and HIVSN subjects respectively. The malaria parasite infection was not statistically significant (P>0.05) among HIVSP and HIVSN (Table: 1).

**Table 1:** Prevalence of malaria parasite in the study populations

Subjects	No. examined	No.(%) infected	No.(%) not infected	% prevalence	X2 cal.	P-value
HIV-seropositive subjects	300	228(76)	72(24)	38	0.34	3.843
HIV-senegative	300	243(78)	66(22)	39		
Total	600	462(77)	138(23)	77		

Table: 2 shows that the mean parasite densities (MPD) of HIVSP subjects was significantly high (P<0.05) when compared with HIVSN subjects. Meanwhile, the mean parasite densities of 2384 ± 747 and 1883 ± 645 were recorded for HIVSP and HIVSN subjects respectively. It was also observed that there was no significant difference (P<0.05) in the prevalence of malaria infection among HIVSP males and females. A mean parasite densities of 2385 ± 782 and 2383 ± 717 were obtained for males and females respectively in this study (Table: 3).

**Table 2:** Mean parasite density of HIV seropositive and HIV seronegative subjects

Subjects	No.	No. (%)	Mean parasite	t cal	P-value
	examined	infected	density (X±S.D)		
HIV seroposite subjects	300	228(76)	2384±747	8.8	1.96
Hiv seronegative subjects	300	234(78)	1883±645		
Total	600	462(77)	2130±740		

**Table 3:** Sex related distribution of malaria parasitaemia amongst HIV seropositive subjects

Sex	No. examined	No. (%) infected	No. (%) not infected	X^2^ cal.	P-value
Males	143	109(76.2)	34(28.8)	0.07	3.841
Female	157	119(75.8)	38(24.2)		
Total	300	228(76)	72(24)		

## Discussion

This study has demonstrated that malaria parasite infection was not significantly different among HIV seropositive and HIV seronegtive (p=3.843)This confirms the earlier report14 that there was no significant difference between HIV infection in adults with parasitaemiaor without to malaria parasite which might be associated with failure to prevent infection as earlier reported^9^.

The overall prevalence rate of 77% observed for malaria parasite infection in this study is far higher than the prevalence rate of 57.9% reported6 in part of Cross River State. This difference may be due to the timing of the study and seasonal variation. The high mean parasite density observed in HIVSP subjects was significantly higher than in HIV and agrees with the earlier report 23 that HIV infection is associated with an increased frequency of malaria parasitaemia.

Meanwhile, the high prevalence rate of malaria parasite infection among HIVSP subjects in this study was far higher than the 7.3% earlier reported^24^ in parts of Delta State. This agrees with the fact that malaria is an endemic disease in Nigeria and constitutes about 60% of illness consultation as earlier reported4. However, this study was carried out partly during the wet season when the vector of malaria parasite (female anopheles mosquito) has a lot of breeding sites.

## Conclusion

This study has shown that there was no significant difference among malaria parasite and HIVSP subjects in the studied area. Therefore, HIV infected patients should be encouraged to avoid malaria infection by ensuring that mosquitoes are adequately prevented by sleeping under insecticide treated net (ITN) as recommended by World Health Organization (WHO) so as to reduce the morbidity and mortality associated with malaria and HIV infections.
